# Can Physical Activity Support the Endocannabinoid System in the Preventive and Therapeutic Approach to Neurological Disorders?

**DOI:** 10.3390/ijms21124221

**Published:** 2020-06-13

**Authors:** Tomasz Charytoniuk, Hubert Zywno, Karolina Konstantynowicz-Nowicka, Klaudia Berk, Wiktor Bzdega, Adrian Chabowski

**Affiliations:** Department of Physiology, Medical University of Bialystok, 15-089 Białystok, Poland; hubert.zywno@gmail.com (H.Z.); karolina.konstantynowicz@umb.edu.pl (K.K.-N.); klaudia.berk@umb.edu.pl (K.B.); wbzdega@gmail.com (W.B.); adrian@umb.edu.pl (A.C.)

**Keywords:** endocannabinoid system, ECS, cannabinoids, phytocannabinoids, physical activity, neurological disorders, neurodegenerative disorders

## Abstract

The worldwide prevalence of neurological and neurodegenerative disorders, such as depression or Alzheimer’s disease, has spread extensively throughout the last decades, becoming an enormous health issue. Numerous data indicate a distinct correlation between the altered endocannabinoid signaling and different aspects of brain physiology, such as memory or neurogenesis. Moreover, the endocannabinoid system is widely regarded as a crucial factor in the development of neuropathologies. Thus, targeting those disorders via synthetic cannabinoids, as well as phytocannabinoids, becomes a widespread research issue. Over the last decade, the endocannabinoid system has been extensively studied for its correlation with physical activity. Recent data showed that physical activity correlates with elevated endocannabinoid serum concentrations and increased cannabinoid receptor type 1 (CB1R) expression in the brain, which results in positive neurological effects including antidepressant effect, ameliorated memory, neuroplasticity development, and reduced neuroinflammation. However, none of the prior reviews presented a comprehensive correlation between physical activity, the endocannabinoid system, and neuropathologies. Thus, our review provides a current state of knowledge of the endocannabinoid system, its action in physical activity, as well as neuropathologies and a possible correlation between all those fields. We believe that this might contribute to finding a new preventive and therapeutic approach to both neurological and neurodegenerative disorders.

## 1. Introduction

The worldwide prevalence of neurological and neurodegenerative disorders has spread extensively in the past two decades among various social groups, races, and ethnicities, becoming a common morbidity and an enormous burden [1,2,3]. Chronic pain, Parkinson’s disease (PD), depression, and anxiety are a tremendous reality with unfavorable prognosis for many individuals. In 2016, the global number of patients who suffered from dementia and Alzheimer’s disease (AD) was about 43.8 million. It increased from 20.2 million in 1990 and will almost double every 20 years, reaching 74.7 million in 2030 and 131.5 million in 2050 [4,5]. Data from numerous studies indicate a substantial correlation between the endocannabinoid system (ECS), a key regulator of energy homeostasis, and the development of neurophysiological processes, such as modulation of neurogenesis, synaptic plasticity, as well as emotions and memory. It was widely demonstrated that the ECS, cannabinoids, and its receptors correlate not only with umpteen physiological aspects, but also are associated with the development of certain neuropathologies, such as pain, depression, as well as neurodegenerative disorders [6,7]. Furthermore, our review will also present a current state of knowledge on the correlation between the endocannabinoid system and physical activity (PA). Although present times are much overwhelmed with a vast amount of research that provides indisputable evidence of the beneficial effects of exercises on neurological pathologies, the exact biological systems and their mechanisms involved in this process are still questionable and widely discussed. Over the last decade, the endocannabinoid system was extensively studied for its correlation with physical activity, which was recognized as a factor that notably modifies this essential biological system and its related molecular pathways [8,9]. Some recent studies demonstrated that plasma levels of endocannabinoids are notably higher after physical activity and might be associated with the long-term beneficial effects on neurophysiology, namely, mood, appetite, mental health, memory, as well as cognitive processes [10,11,12]. Interestingly, some studies indicate that physical activity significantly increases the expression of cannabinoid receptor CB1 (CB1R), a cannabinoid receptor that widely occurs in the striatum, and endocannabinoids, such as anandamide, which both might be correlated with the attenuation of neurological pathologies [10,13]. Several studies revealed that physical activity is associated, at least partially, with its positive results on, for example, metabolic diseases via normalization of the ECS. Furthermore, none of the ECS reviews present a comprehensive correlation between physical activity, its effect on the endocannabinoid system, and subsequently on neurological disorders. Thus, our review significantly contributes to this area of research as it presents many multifaceted aspects of the endocannabinoid system, including its action in physical activity, neurological and neurodegenerative disorders, and a possible correlation between all these fields.

## 2. An Overview of the Endocannabinoid System—From Endo to Phytocannabinoids

The endocannabinoid system is defined as a widespread biological lipid system that plays an essential modulatory role in the endocrine, immune, and brain tissue [14,15]. The endocannabinoid pathway includes elementarily G protein-coupled receptors, known as cannabinoid receptors CB1 and CB2 (CB2R), and the endogenous agonists of these receptors, known as endocannabinoids, principally anandamide (AEA, *N*-arachidonoylethanolamine) and 2-arachidonoylglycerol (2-AG) [16]. It is known that CB1R is predominantly found in the brain (cerebral cortex), whereas CB2R is principally expressed in a number of immune system cells [17]. Considering endocannabinoids, those molecules are synthesized from omega-3 (docosahexaenoic acid, DHA and eicosapentaenoic acid, EPA) or omega-6 (arachidonic acid, AA) long-chain polyunsaturated fatty acids (LC-PUFAs) [18]. Anandamide is a molecule generated from *N*-arachidonoyl phosphatidylethanolamine (NAPE), an AEA membrane precursor, whereas the fatty-acid amide hydrolase (FAAH) catalyzes the hydrolysis of AEA to arachidonic acid and ethanolamine [19]. Although anandamide is a ligand for CB1R, it might interact with non-classic cannabinoid receptors, such as transient receptor potential vanilloid type 1 (TRPV1) or peroxisome proliferator-activated receptors α and γ (PPARα and PPARγ) [20,21]. On the contrary, 2-arachidonoylglycerol, a ligand for both CB1R and CB2R is mainly formed from the degradation of diacylglycerols (DAGs) by diacylglycerol lipases α and β (DAGLα and DAGLβ) and subsequently hydrolyzed by the monoacylglycerol lipase (MAGL) to AA and glycerol [14]. It is worth mentioning that some researchers define an expanded endocannabinoid system as an endocannabinoidome (eCBome)—a complex lipid signaling system composed of more than 100 fatty acid-derived mediators and their receptors, as well as the anabolic and catabolic enzymes of more than 50 proteins. Thus, a vast number of studies reported that eCBome is deeply involved in the control of energy metabolism and its disturbances that may lead to the development of numerous metabolic pathologies [22,23]. Although endocannabinoids constitute a large group of cannabinoids, we may also distinguish two other classes—synthetic cannabinoids and phytocannabinoids—as essential modulators of the endocannabinoid system.

### Phytocannabinoids—Compounds with Dualistic Nature

*Cannabis sativa* L. has been known for both its healing and psychoactive properties for thousands of years. The first reports of pharmacological usage of cannabis came from ancient China, around ~3000 B.C.E., where it was used in the treatment of such conditions as gout, constipation, or rheumatism. In the Medieval Era, *C. sativa* was widely used in the treatment of pain, inflammation, vomiting, and fever. Although the antiepileptic attributes of cannabis were described in the 19th century by Irish physician William O’Shaughnessy, its first use in this pathology was in the Islamic world 100 years prior [24]. Currently, *C. sativa* is the most popular illicit drug in the world, used by approximately 4% of the global population. Still, it is an object of interest to many researchers around the world due to its large potential as a therapeutic agent [25,26]. Phytocannabinoids are a different group of more than 90 terpenophenolic derivatives produced by *C. sativa*. These compounds originate mostly from non-enzymatic reactions of decarboxylation, oxidation, and isomerization of the cannabinoid precursors. In the biosynthesis of phytocannabinoids that occur in *C. sativa* only three enzymes have an essential significance: cannabinoid acid synthase (CBDA), cannabichromenic acid synthase (CBCA), and tetrahydrocannabinolic acid synthase (THCA). These enzymes are responsible for the conversion of a primary phytocannabinoid precursor—cannabigerolic acid (CBGA)—into final products [27,28]. The most abundant component of *C. sativa* is tetrahydrocannabinol (THC), which comprises ~17% of the total phytocannabinoid content and is represented by different isomers, including the most well-known—Δ9-THC. It was proven that Δ9-THC has anticonvulsant, neuroprotective, and anti-inflammatory effects, but its psychoactive and addictive properties cause limitations in its clinical usage. Other phytocannabinoids that may be found in the plant are, for example, cannabidiol (CBD) and cannabinol (CBN) [29,30,31]. The main chemotypes of cannabis preparations are divided into three types: THC predominant (Type I); mixed THC and CBD (Type II, THC and CBD are mixed in 1:1 ratio); and CBD predominant (Type III) [24]. CBD, CBN, and other compounds from this group do not express such psychoactive effects as Δ9-THC [32]. CBD is a much more interesting phytocannabinoid due to its excellent safety profile, and many reported therapeutic effects, especially in the treatment of neurological conditions. Cannabidiol has a very low affinity to cannabinoid receptors. However, it interacts with other complex signaling systems [25,31]. The recent data showed that mechanisms of CBD action might be associated with many molecular targets, including orphan G protein-coupled receptors, serotonin, adenosine, opioid or PPARγ receptors, as well as transient receptor potential (TRP), glycine or sodium channels. CBD also inhibits FAAH which leads to elevation of AEA concentration in serum. The variety of molecular targets for CBD may be correlated with its influence on many different signaling pathways [33]. Furthermore, CBD interacts with cytochrome P450 isoenzymes which may lead to the altered metabolism of other drugs [34].

## 3. Physical Activity and Its Correlation with the Endocannabinoid System and Neurophysiology

### 3.1. PA and the Endocannabinoid System

Physical activity can be defined as repetitive and planned muscle movements that result in energy disbursement. Some researchers broadly portray PA as an essential, cost-saving, and effective factor in terms of prevention, treatment, and management of numerous pathologies [35]. Although the positive effects of physical exercise in the pathophysiology of various diseases are widely known, the molecular mechanisms are still widely discussed. However, one of the most probable and examined mechanisms that is changed during PA is the endocannabinoid system. Physical activity was presented as a significant factor that might lead to the activation of the endocannabinoid-signaling pathway, and a clear mutual correlation between them was indicated in several studies. Based on various research, it is worth noting that PA was demonstrated to modulate the ECS in different ways. Several studies conducted on both animal- and human-based models described significant alterations in blood levels of cannabinoid receptors agonists (i.e., AEA and 2-AG) after exercise. In addition to the endocannabinoids mentioned above, OEA and PEA, the analogous endocannabinoid (eCB) compounds that do not act directly on cannabinoid receptors, were also significantly altered during exercise. Sparling et al. were the first to describe the correlation between acute exercise and higher AEA and 2-AG levels in the blood in human-based models. The elevation of AEA levels may be associated with its acting on peripheral sensory fibers and pain relief as well as the occurrence of “runner’s high” in many regions of the brain, especially in the right anterior lobe and left caudate nucleus [36]. Moreover, the increase in AEA concentration is supposed to be triggered by higher cortisol secretion during acute exercise performance [37,38]. Interestingly, a study by Fuss et al. indicated that exercise-mediated runner’s high might occur due to the interference between physical activity and peripheral CB1 and CB2 receptors, as well as activation of CB1 receptors on forebrain GABAergic (γ-aminobutyric acid) neurons [39]. A large number of studies indicated that moderate and acute physical activity resulted in increased levels of serum concentrations of AEA, OEA, PEA, and 2-AG. Recently, Brellenthin et al. indicated that both AEA and 2-AG circulating levels are significantly higher after exercise, but the increase in AEA is more substantial in the prescribed (approx. 70%–75% max. activity), in comparison to preferred (i.e., self-selected), aerobic exercise [40]. A recent in vivo study conducted by Thompson et al. on mice demonstrated that voluntary physical activity significantly affected circulating endocannabinoid levels differently depending on recent activity and genetic background in comparison to high runner mice (acute PA), which had significantly lower AEA levels. Interestingly, the same study revealed differences in AEA and 2-AG levels between the sexes: males tended to have increased 2-AG levels, whereas AEA levels were higher in females [41]. Recently, Stensson et al. conducted a study on women with fibromyalgia; they indicated that a 15-week person-centered resistance exercise program led to a significant increase in AEA and 2-AG concentration, and therefore might increase muscle strength and provide some neurological alterations, such as analgesia or antidepressant effects [42]. On the contrary, some studies report the constant levels of 2-AG concentration in response to moderate or acute exercises performed by humans [43,44]. Moreover, lower circulating levels of 2-AG after both moderate and preferred physical activity were also demonstrated in some recent research conducted on women with major depressive disorders [11]. Perhaps, alterations in the circulating 2-AG levels depend on the type of physical activity, its intensity, and duration, as well as possible comorbidities. However, further research is necessary to clarify this issue. It is commonly known that physical activity may also affect the expression of cannabinoid receptors, both CB1R and CB2R. Some studies indicated that chronic exercises might be correlated with the upregulation of CB1R expression and density in mice, most notably in the hippocampus [45,46]. Interestingly, a recent study by Crombie et al. revealed that isometric handgrip exercise for three minutes led to significant alterations in the ECS; not only in higher blood circulating levels of AEA, 2-AG, OEA, and PEA, but also increased expression of cannabinoid receptor type 1, which resulted in significant analgesic effects [47]. It is broadly known that the endocannabinoid system and its signaling pathway might be remarkably involved in the dopamine neurotransmission in synapses at midbrain and striatal sites [48]. Furthermore, the activation of cannabinoid receptor type 1 in GABAergic neurons of the human ventral tegmental area (VTA) in the midbrain may result in disinhibition of VTA dopamine release, involved in reward-directed processes that occur during physical activity (mainly voluntary). Thus, this demonstrated a significant and promising correlation between the expression of CB1R, GABA, and dopamine [44,49]. Interestingly, Merill et al. using an animal model indicated that ventral tegmental area GABAergic and DAergic cells are able to produce various eCBs, and therefore might be involved in the alterations in the neuronal activity or plasticity in adaptive reward processing or addiction [50]. These studies might be considered, at least partially, as a way to answer the firm doubts concerning the molecular effects of physical activity on the ECS and higher motivation via the reward system. It is worth mentioning, in terms of physical activity, that stimulation of the CB1R at the nerve terminals of neuromuscular junction might lead to the inhibition of acetylcholine (ACh) release and Ca^2+^ flux that causes decreased muscular tension [35]. Interestingly, there is a lack of findings focused on the interaction between physical activity and phytocannabinoids (e.g., CBD or CBN). Few studies present the action of CBD in muscle recovery by reducing inflammation in the tissue and alleviating pain. However, it underlines the potential usage of phytocannabinoids in the rehabilitation and restoration process after severe physical activity [17,51]. The area of correlation between physical activity and the endocannabinoid system is still unexplored and needs fulfillment by further studies. In addition, novel research should examine the exact mechanism involved in the effect of PA on endocannabinoid signaling, as well as investigate conditions such as the PA type, exercise duration, intensity, age, and sex, which are the most effective in inducing ECS changes.

### 3.2. PA and Neurophysiology—Interference with the Endocannabinoid System

The involvement of physical activity in the neurophysiology components, including mood, pain, cognition, and neurogenesis, is indisputable, as indicated in a vast number of studies. Nevertheless, the mutual relationship between physical activity, the endocannabinoid system, and human neurophysiology remains not well discovered and needs proper fulfillment. The molecular alterations in the endocannabinoid signaling triggered by physical activity mentioned above may directly correlate with systemic effects. Starting with the influence on mood, various types of exercise, both acute and chronic, and ending with resistant and aerobic training, all activate the endocannabinoid signaling and result in significant mood improvements, antidepressant effect, reduced anger, and tension, as well as increased vigor and motivation [35,40,44,52]. Euphoric and analgesic phenomena widely described by athletes that occur during a forced and prolonged physical activity called “runner’s high” are the result of the activity of endorphins, monoamines, and endocannabinoids and their influence on the reward system in the brain [36,53]. Therefore, the “runner’s high” may lead to heavy exercise addiction probably, due to endogenous opioids release, which may be augmented by the ECS [54,55]. On the other hand, the endocannabinoid system is actively involved in the “runner’s high” associated with exercise-induced hypoalgesia. However, the mechanism of this phenomenon is not yet fully understood. Most of the data describe possible interaction between the ECS and the endogenous opioid system in reducing pain sensitivity associated with physical activity. Studies conducted on healthy individuals revealed that short-time isometric exercise produced a significant analgesic effect, which was associated with increased serum concentrations of AEA, 2-AG, and β-endorphins. Moreover, transiently increased pain thresholds in exercising limbs were observed [47,56,57]. On the contrary, a recent study performed by Hughes et al. showed expanded β-endorphin concentrations, whereas 2-AG remained unchanged during forced resistant exercises [58]. The body of evidence suggests that interplay between PA and ECS may influence cognition processes such as memory and learning and may be associated with the development of adult neurogenesis in some regions of the brain. The activation of the HPA (hypothalamic–pituitary–adrenal) axis during stressful situations, which physical activity admittedly is, leads to augmented endocannabinoid synthesis in the peripheral blood and, subsequently, increased activation of postsynaptic β-adrenoceptors which facilitate memory consolidation especially during emotional events [49,59]. Furthermore, a recent article by Wang et al. showed that CB1R signaling in glutamatergic neurons, enhanced by treadmill running, played an essential role in memory and learning improvement and resulted in increased synthesis of neurotrophins and spine density of the hippocampal neurons in mice [60]. Many studies indicate a significant influence of PA and ECS on neurogenesis. Physical activity constitutes a significant factor that leads to enhanced synthesis of BDNF (brain-derived neurotrophic factor), which is a crucial player in the modulation of neurogenesis in the dentate gyrus of hippocampus and subventricular zone. The increased levels of BDNF correlated with expanded AEA and 2-AG levels and CB1R expression within neural progenitor cells. These findings indicate a clear interaction between BDNF and the ECS; what results is the overall promotion of proliferation, regeneration, and viability of neurons [61,62,63]. Interestingly, Heyman et al. showed that intense and prolonged physical activity resulted in enhanced synthesis of BDNF among male cyclists, probably due to elevated circulating levels of endocannabinoids (i.e., AEA among male cyclists). These findings correlate with neuroplasticity development and antidepressant effect induced by exercise [37]. In summary, the activation of the endocannabinoid system through various types of physical activity may provide a promising influence on neurophysiology aspects. The popularity of physical activity as a cheap, plausible, and effective method of maintaining a healthy lifestyle and prophylaxis of an enormous number of disorders, including neurological, is increasing tremendously worldwide. Therefore, we believe that more data focused on positive outcomes of different types of exercise may even increase awareness among people and, at least partially, contribute to decreased prevalence of neurological and neurodegenerative conditions. The large number of studies conducted both on animal- and human-based models shows that targeting the ECS by physical activity may provide promising results in the treatment of neurological conditions; they have been gathered and examined and are presented in Table 1.

## 4. The Endocannabinoid System and Its Correlation with Neuropathologies

The endocannabinoid system constitutes a crucial player in the development of various neuropathological states, including depression, Alzheimer’s disease, Parkinson’s disease, multiple sclerosis, and epilepsy. These conditions probably may also be caused by dysregulations that occur in the endocannabinoid signaling pathway. The background of these disturbances seems to be very complex and includes altered cannabinoid receptors signaling and expression as well as fluctuations in endocannabinoid concentrations in serum [70,71].

### 4.1. Depression and Anxiety

Significant alterations in cannabinoid receptors expression occur in depression and anxiety. The genetic overexpression of the CB2R resulted in decreased depressive-like behavior, whereas CB1R deficiency was correlated with the development of depressive symptoms in rodents. In human studies, patients with depression had lower levels of AEA and 2-AG in serum [72,73,74]. Studies conducted on the brains of patients with depression who committed suicide showed an increased density of CB1R in the prefrontal cortex. The density of CB2R remained unchanged [75]. This evidence suggests that the ECS hypoactivity may result in the development of depression and depression-like states. Certain polymorphisms in the CB1R coding gene—*CNR1*—seem to be associated with susceptibility to depression and its treatment-resistance development. In turn, the knockout of the CB1R in mice resulted in anxiety-like behavior [72,76,77]. Moreover, a study conducted by Kong et al. revealed that certain polymorphisms in *CNR2* coding CB2R also correlated with increased susceptibility to depression development among patients [78]. Furthermore, overexpression of CB1R was detected in anxiety-related brain areas such as the amygdala, hippocampus, and striatum among posttraumatic stress disorder (PTSD) diagnosed patients [75]. Additionally, decreased levels of AEA and 2-AG were described in the blood of patients with PTSD [79].

### 4.2. Alzheimer’s Disease (AD)

In the animal models that expressed a mutant form of amyloid precursor protein (APP), including Tg2576 transgenic mice and APP/PS1 mice, significant alterations in the ECS were observed. Most of the studies showed the downregulation and impaired signaling within CB1R in microglial cells of the hippocampus and prefrontal cortex of transgenic mice [80,81,82]. In turn, the upregulation of CB2R that occurred in mice models may be associated with neuroprotective and anti-inflammatory properties resulting from CB2R activation [70,81]. The higher serum levels of 2-AG were associated with hippocampal degradation induced by amyloid-β peptide [70,80]. Altmura et al. revealed that the elevated levels of 2-AG might be associated not only with neuroprotective mechanisms but also with amelioration in cerebral circulation [83]. Furthermore, overexpression of FAAH was revealed in the brains of people with AD and may correlate with exacerbated inflammatory processes [80,84,85]. The results of the clinical trials or post-mortem studies also showed elevated levels of the endocannabinoids and higher expression of CB2R. However, the expression changes in CB1R remained inconsistent. Some studies showed no alterations in the expression and density of CB1R. In contrast, a significant decrease in the expression of this receptor in the brain cortex of AD patients was found. Thus, these contradictory data should be clarified by future research [81,84].

### 4.3. Parkinson’s Disease (PD)

In Parkinson’s disease, considerable changes within the ECS were observed and well described in studies conducted on animal models as well as shown in the post-mortem brains of PD patients. These changes included both hyper- and hypoactivity of CB1R signaling, increased levels of 2-AG and AEA, and altered expression of CB1R and CB2R in the basal ganglia of people with PD and transgenic mice including 1-methyl-4-phenyl-1,2,3,6-tetrahydropyridine (MPTP)-lesioned mouse models and lipopolysaccharide (LPS) rat models [85,86,87]. A recent study conducted on brain samples of patients with PD revealed a significant decrease in MAGL expression [88]. Ceccerini et al. showed that individuals with PD-related cognitive impairment had decreased expression of CB1R, especially in the midcingulate and superior frontal gyrus [89]. Furthermore, this receptor is supposed to be involved in motor disturbances that occur in PD. The administration of CB1R antagonists, such as rimonabant, resulted in ameliorated dyskinesia and motor impairment in experimental models (i.e., 6-hydroxydopamine (6-OHDA) or MPTP-lesioned rats) [90]. The upregulation of CB2R may be associated with the anti-inflammatory process and reduction of the degradation of dopaminergic neurons, whereas the downregulation of these receptors results in exacerbation of these processes [80,85].

### 4.4. Multiple Sclerosis (MS)

Studies conducted on mice with experimental autoimmune encephalomyelitis (EAE)—a preclinical model of MS—showed that complex alterations in CB1R and CB2R signaling occur. The activation of these receptors was correlated with neuroprotective and anti-inflammatory effects [86]. The CB1R and CB2R knockout mice showed enhanced inflammation and reduced neurodegeneration [91]. In turn, the concentration of AEA was significantly increased, whereas, 2-AG remained unchanged or either increased in mice with EAE [48,87]. Human studies revealed significant elevation of serum AEA, OEA, and PEA concentrations. As in the animal data, 2-AG remained unchanged. Furthermore, the levels of anandamide were increased in the cerebrospinal fluid (CSF), brain, and peripheral tissues of MS patients. The mRNA of both CB1R and CB2R was increased among patients with primary-progressive MS which might suggest possible compensating mechanisms [92,93,94]. Moreover, an increased expression and activity of FAAH were detected [95]. These findings show that the ECS is involved in multiple sclerosis pathogenesis and targeting the ECS may be a promising treatment method for relapsing or acute multiple sclerosis.

### 4.5. Epilepsy

Interestingly, there is a lack of data that describes the involvement of the ECS in epilepsy pathogenesis. In most preclinical studies, the injection of kainic acid or pentylenetetrazole, as well as an electric shock, were used to induce an acute seizure attack in animal models. In the studies conducted on animals, overexpression of CB1R, and elevated concentrations of 2-AG in blood after induction of seizures were described [96,97]. A significant decrease in expression of CB1R and DAGL-α was revealed in the human epileptic hippocampus. Moreover, the concentration of AEA in the cerebrospinal fluid of epileptic patients was also decreased. All these changes were associated with impaired GABA signaling in the brain [96,98]. Nowadays, the usage of cannabinoid-derived compounds in the treatment of epilepsy, especially the drug-resistant kind, is becoming more common worldwide. The majority of studies showed notable relieving effects after cannabis treatment on epilepsy management, mainly drug-resistant ones [99]. Jessberger et al. described a possible correlation between seizure-generated new granular cells and the promotion of neurogenesis in the adult brain, particularly in the hippocampus in what may be a compensating mechanism of “brain recovering” after an injury caused by seizure [100].

## 5. The Triad—Physical Activity, the Endocannabinoid System, and a Novel Therapeutic Approach to Neurological Pathologies—How Might All These Be Linked?

**Figure 1:**
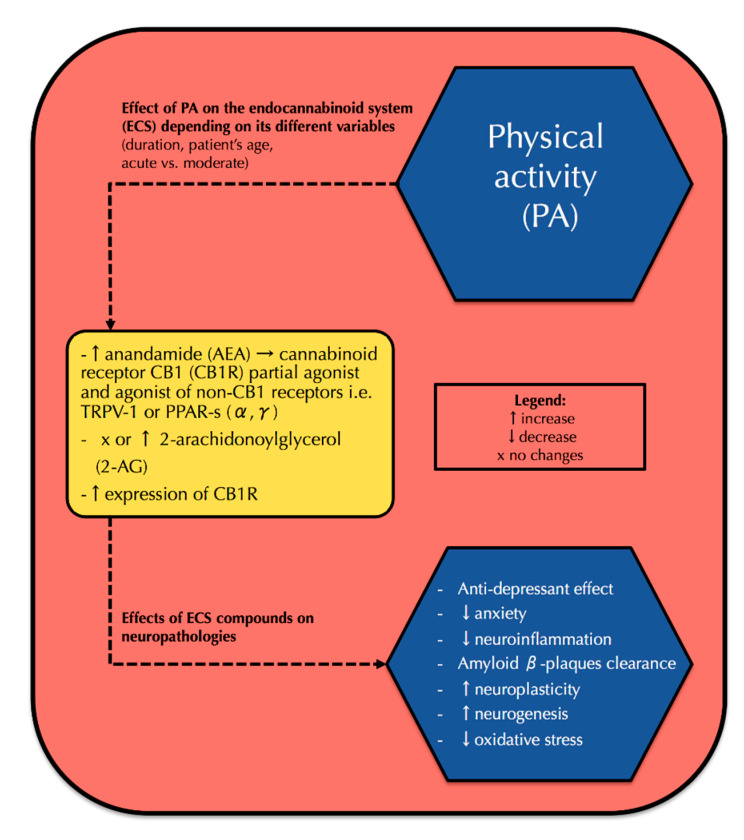
The effect of physical activity on the endocannabinoid system components and, subsequently, its possible impact on the attenuation of neuropathologies. PA: physical activity, ECS: endocannabinoid system, AEA: anandamide, CB1R: cannabinoid receptor type 1, TRPV-1: transient receptor potential vanilloid type 1, PPAR: peroxisome proliferator-activated receptors α, 2-AG: 2-arachidonoylglycerol.

The summary of the correlation between physical activity, its effect on the endocannabinoid system, and subsequently neuropathologies is presented in Figure 1.

There is no doubt that the involvement of the ECS in the pathogenesis of both neurological and neurodegenerative disorders is crucial. The targeting of many ECS components, including cannabinoid receptors, endocannabinoids, or enzymes responsible for their degradation by both natural and synthetic agents, may be a promising and effective treatment method for these conditions and accompanying symptoms. However, despite some studies, there is still a number of undiscovered areas in this field. Furthermore, a broad correlation between the endocannabinoid system and physical exercises, both acute and chronic, was indicated in a number of both animal- and human-based models [8,35]. Understanding the effects of physical activity on the ECS may contribute towards considering exercise as an alternative approach to the clinical management of neuropathologies. Although there is a limited amount of evidence demonstrating positive ECS-related effects of physical activity on the attenuation of, for example, obesity and type 2 diabetes mellitus (T2DM), the involvement of physical activity in the treatment of neurological diseases by affecting the endocannabinoid signaling is not yet well discovered [38,101,102]. Interestingly, studies conducted on animal models after one weak treadmill running revealed ameliorated spatial memory results in a common memory test with an increased CB1R expression in the hippocampus [46]. Furthermore, the activation of the ECS by physical activity is widely considered to suppress inflammation and oxidative stress in neuronal tissue by affecting cannabinoid receptors signaling and modulating the function of lymphocytes [35,44]. Thus, targeting the ECS by physical activity might provide auspicious treatment results of various neurological disorders. We believe that this uncharted territory with excellent research potential may constitute a field that will be broadly explored within the next few years. Moreover, physical activity will be considered, at least partially, as a novel therapeutic approach to the attenuation of neuropathologies.

## 6. Conclusions

In conclusion, our review comprehensively summarizes substantial studies that describe precisely the endocannabinoid system from its molecular structure and compounds to its correlation with physical activity. Moreover, we profoundly demonstrate the ECS action in various neurological and neurodegenerative pathologies, as well as point out mechanisms that might be investigated in further studies. The development and worldwide prevalence of neuropathologies are very alarming, and their prevention has become a substantial issue. Therefore, it is essential to find new therapeutic targets, which may probably contribute to a reduction in the annual increase of individuals suffering from these diseases among all of the global populations. Consequently, researchers have great hope for the endocannabinoid system, its elements, pathways, and ligands, and consider the ECS as a possible preventive and therapeutic approach for both neurological and neurodegenerative pathologies, which might be correlated, at least partially, with physical activity.

## Figures and Tables

**Table 1:** A summary of studies analyzing the correlation between physical activity, the ECS, and possible positive outcomes on brain physiology and various neurological disorders.

Subjects	Performed Activity	Main Outcomes	Reference
Healthy men runners (*n* = 8), cyclist (*n* = 8), controls (*n* = 8)	Running on a treadmill/cycling on an ergometer for 45 min (HR_max_ = 70%–80%)	ECS alterations: ↑AEA	[36]
		Brain physiology and neurological alterations: Anxiolytic and analgesic effect, sense of well-being → “runner’s high”	
Well trained male cyclist (*n* = 11)	Moderate cycling on an ergometer for 60 min (55% W_max_) followed by intense cycling for 30 min (75% W_max_)	ECS alterations: ↑AEA, PEA, OEA	[37]
		Brain physiology and neurological alterations: Increased BDNF and cortisol levels, antidepressant and reward effect, possible promotion of neuroplasticity	
Women with fibromyalgia (*n* = 37), controls (*n* = 33)	15-week person-centered resistance exercise program	ECS alterations: ↑AEA, 2-AG	[42]
		Brain physiology and neurological alterations: Antidepressant and analgesic effect, increased muscle strength	
Patients with PTSD (*n* = 12), controls (*n* = 24)	Low/moderate 10 min warm-up (HR_max_ = 40%–60%) followed by 30 min of moderate walking or running on a treadmill (HR_max_= 70%–75%).	ECS alterations: ↑AEA, 2-AG, OEA	[64]
		Brain physiology and neurological alterations: Antidepressant effect, analgesic effect, reduced stress, fatigue, confusion, anger, and anxiety	
Patients with episodic migraine (*n* = 30), controls (*n* = 28)	12 week aerobic exercise program—40 min of walking/running on a treadmill 3 times per week	ECS alterations: ↓AEA	[65]
		Brain physiology and neurological alterations: Amelioration of migraine headaches, reduced frequency of migraine attacks	
Women with MDD (*n* = 17)	30 min of moderate cycling followed by 30 min of preferred exercise	ECS alterations: ↑AEA, OEA, ↓2-AG	[11]
		Brain physiology and neurological alterations: Minimal antidepressant effect	
Patients with relapsing-remitting MS (*n* = 30)	2 weeks of therapeutic exercise program—1 h of aerobic exercise followed by 1 h of swimming in the pool.	ECS alterations: Different polymorphisms in *CNR1* gene lead to various responses on physical therapy associated with altered CB1R density in motor cortex.	[66]
		Brain physiology and neurological alterations: ↑ cortical plasticity and response to physiotherapy	
Healthy men (*n* = 29) and women (*n* = 29)	Isometric handgrip exercise for 3 min (MVC = 25%)	ECS alterations: ↑AEA, 2-AG, OEA, PEA ↑CB1R	[47]
		Brain physiology and neurological alterations: Significant analgesic effect; ECS interplays with endogenous opioid release → “exercise-induced antinociception”	
Healthy women (*n* = 9)	1 day—30 min of dancing 2 day—30 min of cycling on ergometer	ECS alterations: ↑OEA (only while dancing)	[10]
		Brain physiology and neurological alterations: Reduced appetite, decreased negative emotions, “runner’s high”	
Cannabis users (*n* = 37), controls (*n* = 42)	Treadmill running	ECS alterations: not described	[67]
		Brain physiology and neurological alterations: Improved psychomotor speed, visual memory, sequencing ability among cannabis users → possible interplay between cannabinoids and physical activity	
Male Sprague-Dawley rats (*n =* 40)	Wheel running	ECS alterations: ↑AEA, CB1R	[61]
		Brain physiology and neurological alterations: Increased progenitor cell proliferation within dentate gyrus, promotion of neurogenesis	
Male Wistar rats treated with LPS (animal model presenting signs of neuroinflammation)	Forced treadmill running for 8 weeks 5 times per week. MWT performed.	ECS alterations: ↑2-AG, CB1R	[68]
		Brain physiology and neurological alterations: Improved memory and cognitive function, reduced inflammatory effect ↓COX-2	
Male Swiss mice (*n* = 72)	5 min of treadmill running for 3 days	ECS alterations: ↑CB1R	[46]
		Brain physiology and neurological alterations: Increased spatial memory ↑BDNF	
Male Swiss mice	High-intensity swimming exercise (HISE)	ECS alterations: ↑AEA, CB1R	[69]
		Brain physiology and neurological alterations: Significant analgesic effect → “exercise-induced antinociception”, reduced inflammation	

S2-AG: 2-arachidonoylglycerol, AEA: anandamide, BDNF: brain derived neurotrophic factor, CB1R: cannabinoid receptor type 1, ECS: endocannabinoid system, HR_max_: maximum heart rate, MDD: major depressive disorder, HISE: high-intensity swimming exercise, MS: multiple sclerosis, MVC: maximum ventilatory capacity, MWT: maze water test, OEA: *N*-oleoylethanolamine, PEA: palmitoylethanolamide, W_max_: maximal trial power output, LPS: lipopolysaccharide. ↑—increase, ↓—decrease, →—further step.

## Abbreviations

2-AG	2-arachidonoylglycerol
6-OHDA	oxidopamine
AA	arachidonic acid
ACEA	arachidonyl-2-chloroethylamide
ACEA	arachidonyl-2-chloroethylamide
ACh	acetylcholine
AD	Alzheimer’s disease
AEA	anandamide, N-arachidonoylethanolamine
Aβ	amyloid-β
BACE1	β-secretase 1
BDNF	brain-derived neurotrophic factor
CB1R	cannabinoid receptor type 1
CB2R	cannabinoid receptor type 2
CBCA	cannabichromenic acid synthase
CBD	cannabidiol
CBDA	cannabinoid acid synthase
CBGA	cannabigerolic acid
CBN	cannabinol
CSF	cerebrospinal fluid
DA	dopamine
DAG	diacylglycerol
DAGLα	diacylglycerol lipase α
DAGLβ	diacylglycerol lipase β
DHA	docosahexaenoic acid
EAE	autoimmune encephalomyelitis
eCB	endocannabinoid
eCBome	endocannabinoidome
ECS	endocannabinoid system
EHA	eicosapentaenoic acid
EPM	elevated plus maze test
FAAH	fatty-acid amide hydrolase
FST	forced swimming test
GABAHPA	γ-aminobutyric acidhypothalamic–pituitary–adrenal
HISE	high-intensity swimming exercise
HR_max_	maximum heart rate
LC-PUFAs	long-chain polyunsaturated fatty acids
LPS	lipopolysaccharide
MAGL	monoacylglycerol lipase
MDD	major depressive disorder
MPTP	1-methyl-4-phenyl-l,2,3,6-tetrahydropyridine
MS	multiple sclerosis
NAPE	*N*-arachidonoyl phosphatidylethanolamine
OEA	*N*-oleoylethanolamine
PA	physical activity
PD	Parkinson’s disease
PEA	palmitoylethanolamide
PPARα	peroxisome proliferator-activated receptors α
PPARγ	peroxisome proliferator-activated receptors γ
T2DM	type 2 diabetes mellitus
THCA	tetrahydrocannabinolic acid synthase
TRP	transient receptor potential
TRPV1	transient receptor potential vanilloid type 1
VTA	ventral tegmental area
Δ9-THC	Δ9-tetrahydrocannabinol
MVC	maximum ventilatory capacity
W_max_	maximal trial power output
